# Purification and Characterization of Lipase Produced by *Leuconostoc mesenteroides* Subsp. *mesenteroides* ATCC 8293 Using an Aqueous Two-Phase System (ATPS) Composed of Triton X-100 and Maltitol

**DOI:** 10.3390/molecules23071800

**Published:** 2018-07-20

**Authors:** Nurfadhilah Hidayah Eko Sukohidayat, Mohammad Zarei, Badlishah Sham Baharin, Mohd Yazid Manap

**Affiliations:** 1 Faculty of Food Science and Technology, Universiti Putra Malaysia, 43400 Serdang, Selangor, Malaysia; mzarei.mail@gmail.com (M.Z.); badli@upm.edu.my; (B.S.B.); yazidmanap@icloud.com (M.Y.M.); 2 Department of Food Science and Technology, College of Agriculture and Natural Resources, Sanandaj Branch, Islamic Azad University, 66131 Sanandaj, Iran; 3 Halal Product Research Institute, Universiti Putra Malaysia, 43400 Serdang, Selangor, Malaysia

**Keywords:** aqueous two phase system, maltitol, Triton X-100, lipase, *Leuconostoc mesenteroides*, characterization

## Abstract

Purification of lipase produced by *L. mesenteroides* subsp. *mesenteroides* ATCC 8293 was conducted for the first time using a novel aqueous two-phase system (ATPS) composed of Triton X-100 and maltitol. The partitioning of lipase was optimized according to several parameters including pH, temperature, and crude load. Results showed that lipase preferentially migrated to the Triton X-100 rich phase and optimum lipase partitioning was achieved in ATPS at TLL of 46.4% and crude load of 20% at 30 °C and pH 8, resulting in high lipase purification factor of 17.28 and yield of 94.7%. The purified lipase showed a prominent band on SDS-PAGE with an estimated molecular weight of 50 kDa. The lipase was stable at the temperature range of 30–60 °C and pH range of 6–11, however, it revealed its optimum activity at the temperature of 37 °C and pH 8. Moreover, lipase exhibited enhanced activity in the presence of non-ionic surfactants with increased activity up to 40%. Furthermore, results exhibited that metals ions such as Na^+^, Mg^2+^, K^+^ and Ca^2+^ stimulated lipase activity. This study demonstrated that this novel system could be potentially used as an alternative to traditional ATPS for the purification and recovery of enzymes since the purified lipase still possesses good process characteristics after undergoing the purification process.

## 1. Introduction

Recently, the demand for enzymes to be used in different related industries is growing, however the high cost of enzymes is still a major concern for the most of them. Enzymes are costly due to the complexity and difficulty of their downstream processing as they usually involve various purification steps that account for more than 70% of total production cost of the enzymes [1]. The purification methods of enzymes traditionally used in the industries include several steps such as ultrafiltration, precipitation, and chromatography, which might result in the loss of product in each step [2]. Furthermore, these conventional methods of purification, which includes the preparation of lipases for further purification, chromatographic steps, and membrane processes, are expensive, multisteps, and discontinuous as well as they are labor and time extensive.

Aqueous two-phase system (ATPS) is an alternative purification technique that was introduced during the mid-1950’s by Albertsson [3]. However, its potential as an efficient purification method has been realized, recently. ATPS possess several interesting advantages including economical, continuous, simple, and rapid as the clarification, concentration and purification of the sample of interest could be accomplished in a single step. Besides, ATPS serves as a friendly and gentle environment for biological materials due to its water-rich environment. Generally, ATPS based on polymer/polymer and polymer/salt are the most common systems used in the purification of biomolecules. However, these two traditional systems have some drawbacks such as the use of salt in the system might lead to the denaturation or certain target proteins may precipitate due to the high ionic-strength environment [4]. Furthermore, additional laborious purification techniques, such as ultrafiltration, diafiltration and crystallization are necessary for the removal of phase-forming chemicals/polymers from the target protein [5]. In order to overcome the weaknesses of the traditional ATPS systems, new ATPS systems composed of non-ionic surfactants and polyols have been discovered and explored [6,7]. When non-ionic surfactants are used as a component in ATPS, the system will separate into two phases, in which one of the phases will be the surfactant-depleted phase (water-rich phase) whereas the other phase will be the surfactant-rich phase. Triton X-100 is favorable for the purification of proteins as it can solubilize proteins in a gentle fashion, hence, the target proteins will not lose their biological activity at the end of a purification stage. In recent years, ATPS composed of polyols have received increasing attention in the purification studies. Polyols like maltitol, which are also known as sugar alcohols, are a hydrogenated form of carbohydrates and their carbonyl group has been reduced to a primary or secondary hydroxyl group. They are able to mimic the structure of water; therefore, they are able to form and maintain an artificial sphere of hydration around the target biomolecules [6].

Lipases (triacylglycerol acylhydrolase E.C. 3.1.1.3) are a group of enzymes that are able to catalyze the hydrolysis and synthesis of triglycerides from glycerol and free fatty acids [8,9]. Although there are many lipases currently available, they are still insufficient to fulfill the operating requirements of most industries as lipases produced by different microbes have different optimal reaction conditions. These conditions can greatly influence the applications of lipases in the industries. Hence, enzyme characterization is crucial to determine their suitability in a particular industrial process.

Although there are many reports on the purification of lipase from Lactic Acid Bacteria (LAB) [10,11,12], to date there is no report on the purification of lipase from these bacteria using ATPS. One of the bacterium in the LAB group, *L. mesenteroides*, is a non-pathogenic and classified as generally recognized as safe (GRAS) organism by the U.S Food and Drug Administration (FDA). These characteristics are essential for the commercial applications of lipases in various industries, particularly in food industry. Thus, in this study, lipases obtained from *L. mesenteroides* subsp. *mesenteroides* ATCC 8293 were used for purification and characterization.

To the best of our knowledge, there is currently no report on the purification of lipase from *L. mesenteroides* subsp. *mesenteroides* ATCC 8293 using ATPS composed of Triton X-100 and maltitol. This is the first time that Triton X-100 and maltitol have been proven to undergo phase separation and used as a purification technique. In addition, lipase from *L. mesenteroides* subsp. *mesenteroides* ATCC 8293 was also characterized for the first time. In the present study, the binodal curve and Tie-Line Length (TLL) of the novel ATPS was constructed and developed as well. Additionally, the efficiency of lipase partitioning in the ATPS and the effects of crude feedstock, pH and temperature were investigated in order to achieve a high lipase yield and finally the characteristic properties of the purified lipase were determined.

## 2. Results and Discussion

### 2.1. Effect of Triton X-100 and Maltitol on Lipase Activity

In this work the supernatant containing the crude lipase produced by *L. mesenteroides* subsp. *mesenteroides* ATCC 8293 was directly used for the purification study using the ATPS composed of Triton X-100 and maltitol after centrifugation. In order to determine the compatibility of the phase-forming components with the enzymes, the effect of different concentrations of these components on the lipase activity was investigated at 25 °C for 1 h. The results (Table 1) demonstrated that increase in Triton X-100 concentrations led to the increase in the lipase activity. It has been reported that non-ionic surfactant such as Triton X-100 induces lipase activity, and therefore, reduces enzyme denaturation. Besides, Triton X-100 could also increase enzyme activity by affecting the enzyme-substrate interaction, in which, the addition of this non-ionic surfactant prevented the adsorbed enzyme from inactivation [5,7]. However, at 50% (*w*/*w*) Triton X-100 concentration, lipase activity started to decrease as higher concentrations of surfactants negatively affected the amount of solubilized enzyme and its catalytic action. Mehrnoush et al. [5,7] have reported that the partitioning of enzymes in the presence of high concentrations of Triton X-100 was reduced in ATPS as the stability of the targeted enzymes significantly decreased in the presence of the high concentrations of surfactants.

### 2.2. Binodal Curve and TLL

**Figure 1:**
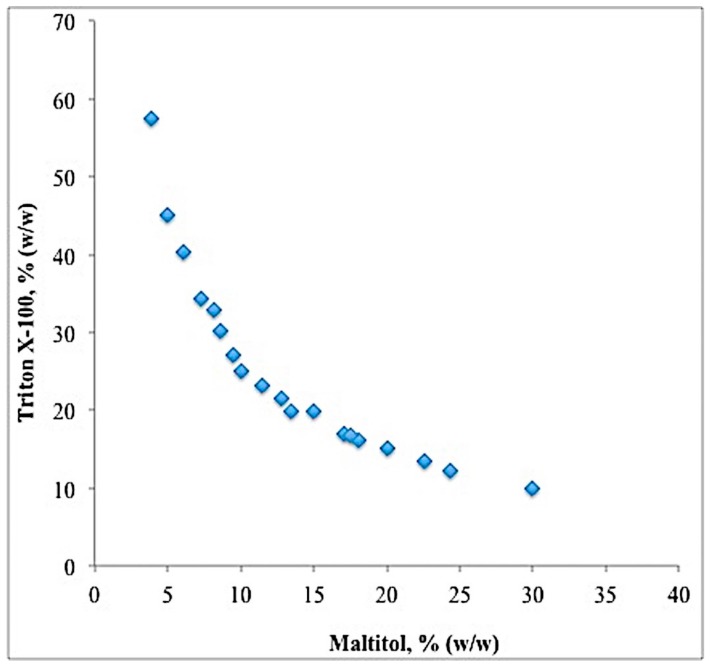
Binodal curve for ATPS composed of Triton X-100, maltitol and water at room temperature.

The experimental binodal curve for both Triton X-100 and maltitol were determined at room temperature using turbidometric titration method. In this work, it was observed that the bottom phase is maltitol-rich phase whereas the top phase is Triton X-100-rich phase. The data obtained by this method were then used to construct a binodal curve in the phase diagram. In Figure 1, the maltitol-rich bottom phase is plotted as the abscissa whereas the Triton X-100-rich top phase is plotted as the ordinate.

The experimental binodal data were fitted using the empirical relationship as described by Equation (1). Subsequently, several TLLs were calculated. The TLLs were obtained using the gravimetric method [13]. The values of TLLs were presented in Table 2. Based on Table 2, maximum lipase partitioning at 46.4% (*w*/*w*) TLL was observed, with lipase selectivity of 10.25 and lipase purification factor of 7.41. It should be noted that the increase in purification factor of 7.42 at TLL of 46.4% (*w*/*w*) was mainly resulted by the removal of the contaminant proteins.

Maximum lipase partitioning at TLL of 46.4% (*w*/*w*) indicated that the fine balance between the Triton X-100 and maltitol was achieved. The increase in the TLL led to higher concentration of maltitol in the bottom phase. Maltitol is a non-ionic compound with an enhanced ability to be hydrated due to its large number of –OH groups, which makes it a strong phase separating agent. Due to its strong phase separating characteristics, maltitol improves the migration of lipase to the opposite phase [6], which was Triton X-100-rich top phase, hence, increasing the efficiency of the system during lipase partitioning process.

### 2.3. Effect of Crude Feedstock on Lipase Partitioning

**Figure 2:**
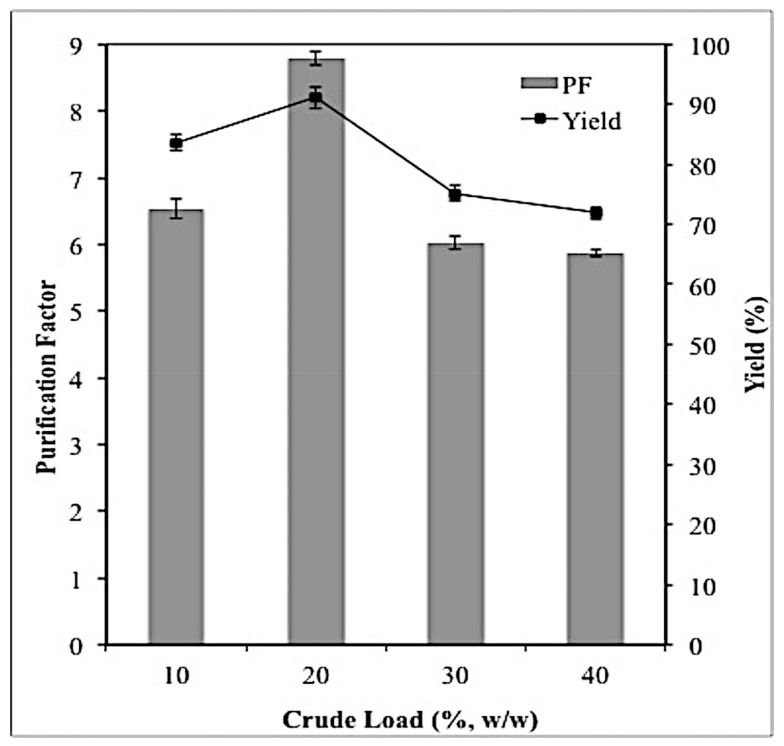
Effect of crude load on lipase partitioning. Lipase purification factor and yield were calculated using Equations (11) and (12), respectively. All the assays were conducted in triplicate and the average values were taken.

The addition of different amounts of crude feedstock to the ATPS will influence the partitioning ability of the system on the targeted enzyme as the feedstock load may alter the two-phase volume ratio of the system as well as the partition behavior of the enzyme of interest [14]. As shown in Figure 2, increasing the crude load from 10% to 20% (*w*/*w*) showed the maximum capacity for efficient purification of lipase as it resulted in the highest purification factor (8.79) and yield (91.1%) compared to the other feedstock concentrations. It was also observed that the partitioning efficiency of the ATPS decreased when the concentrations of the crude feedstock were higher than 20% (*w*/*w*). Higher amounts of crude feedstock, which were 30% and 40% (*w*/*w*), negatively impacted the partitioning ability of the ATPS because they can alter the properties of the system by reducing the volume ratio of the two phases, which then lead to the changes in the composition of top and bottom phases of the system.

10% (*w*/*w*) crude load showed better results than those of 30% and 40% (*w*/*w*) because it did not affect the composition of the ATPS components since the concentration of 10% (*w*/*w*) crude load did not exceed the concentration limit. Hence, the partitioning behaviors of the target enzyme remained unchanged. On the other hand, the crude load of 30% and 40% (*w*/*w*) exceeded the concentration limit, which was 20% (*w*/*w*). Due to this, both crude load concentrations altered the partition behavor of the the target lipase. Therefore, instead of being partitioned into the top phase, the target protein was found to be precipitated at the interface due to the lack of space to accommodate the target enzyme in the top phase, which then led to the loss of lipase together with other contaminants in the purification process [7]. Therefore, a crude feedstock of 20% (*w*/*w*) was the optimum concentration of feedstock for lipase partitioning and purification. Similarly, purification of lipase from *Burkholderia cenocepia* strain 8 by using ATPS composed of ethylene oxide-polypylene oxife (EOPO) and potassium phosphate required a crude load of 20% (*w*/*w*) in order to achieve maximum recovery [14]. In contrast, maximum recovery of *Burkholderia cepacia* lipase was reported when a crude load of 35% (*w*/*w*) was used in the ionic liquid/polymer-based ATPS [4].

### 2.4. Effect of PH on Lipase Partitioning

**Figure 3:**
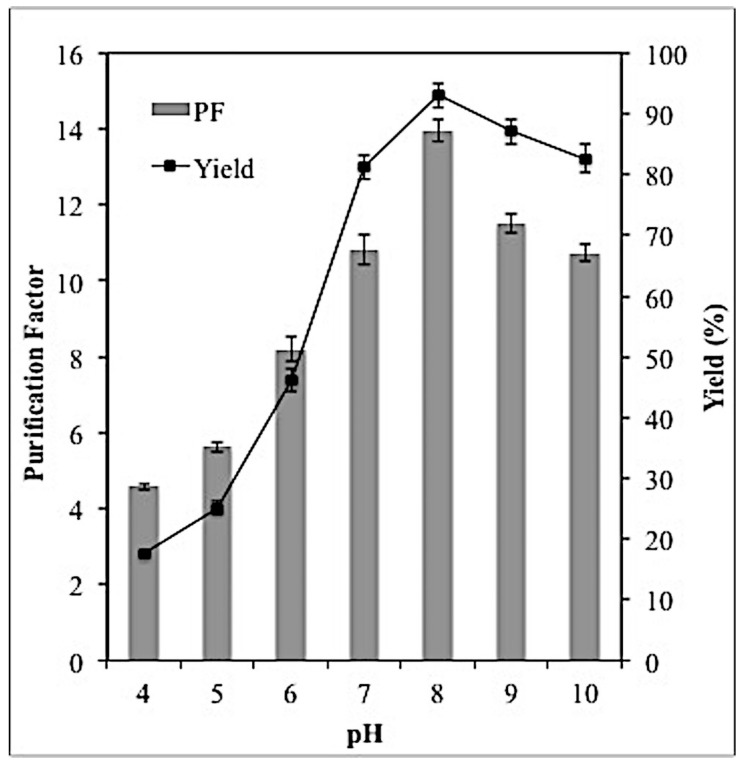
Effect of various pH on lipase partitioning in the top phase was investigated using 46.4% (*w*/*w*) TLL and 20% (*w*/*w*) crude load. All the assays were conducted in triplicate and the average values were taken.

The effect of pH on the selectivity and yield of lipase was illustrated in Figure 3. Based on the results, higher lipase yield was observed in the pH range 7–10 whereas in the acidic pH range (4–6), the yield of lipase was severely reduced. Similar to the yield, the selectivity of lipase in the acidic conditions (pH 4–6) was significantly lower compared to neutral and alkaline pH. The pH of the system has immense influence on lipase partitioning as it can either alter the ratio of the charged molecules or the charge of the solute. Lipase has a p*I* about 6.3 [14], therefore when the pH of the system was below the p*I* (pH 4–6), the lipase was in its cationic form, in which it was highly soluble in water, hence, it moved to the bottom phase. However, at pH 7–10, the targeted lipase tends to be negatively-charged and partitioned into the hydrophobic region, which was Triton X-100-rich top phase whereas the other contaminants were partitioned into the maltitol-rich bottom phase.

This result is in agreement with several researchers who have revealed that at higher pH, the negatively charged protein prefers to partition into the top phase of the system [1,5,7,14], which in turn increase the partition coefficient as well as the purification factor of the lipase. As shown in Figure 3, the PF and yield of the lipase were significantly reduced when the pH was higher than 8.0. This might be due to the decrease in lipase activity at pH values higher than 8. Thus, the maximum purification factor (13.96) and yield (93%) of the lipase were observed at pH 8. Similar finding was observed by Mehrnoush et al. [5] who reported that lipase extracted from *Cucurbita moschata* seeds demonstrated maximum partitioning ability at pH 8 using Triton X-100/maltitol ATPS. However, *Burkholderia cenocepia* strain 8 lipase exhibited maximum partitioning ability at pH 7 in an ATPS composed of ethylene oxide-polypylene oxide (EOPO) and potassium phosphate [14].

### 2.5. Effect of Temperature on Lipase Partitioning

**Figure 4:**
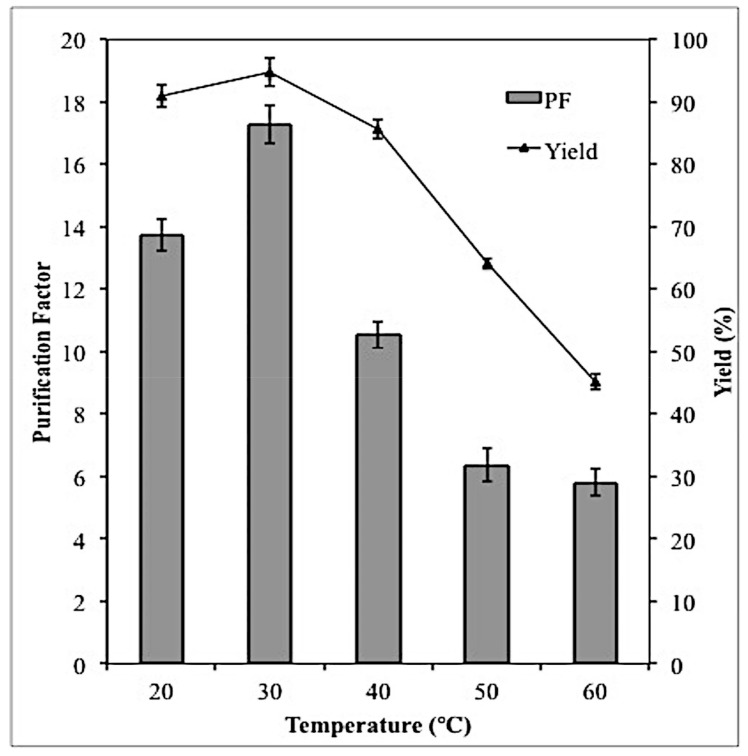
Effect of different temperatures on lipase partitioning in the top phase was investigated at 46.4% (*w*/*w*) TLL, 20% (*w*/*w*) crude load and pH 8. All the assays were conducted in triplicate and the average values were taken.

The temperature of the ATPS can influence the lipase partitioning by either altering the structure of the lipase or causing it to be denatured. According to Figure 4, increase in lipase selectivity and yield could be observed between 20–30 °C Beyond 30 °C, lipase selectivity and yield decreased strongly with increasing temperature. In general, when proteins are exposed to increasing temperature, a number of bonds in the protein molecule become weak and at certain point they may be broken.

At elevated temperature, some of the hydrogen bonds in the protein molecule that are responsible to stabilize the helical structure of the protein might be broken [15]. Due to this, water molecules in the system will interact and form new hydrogen bonds with the amide nitrogen and carbonyl oxygen of the peptide bonds whereas the hydrophobic groups of the protein will be exposed to the hydrophobic groups of the Triton X-100. At this point, the protein has completely denatured, as it cannot refold back to its native structure, hence, the protein loses its solubility and its enzymatic activity, which then resulted in low lipase yield and selectivity. In this work, maximum purification factor (17.28) and yield (94.7%) was obtained at 30 °C. On the contrary, Carvalho et al. [16] reported that the best temperature for the purification of *Yarrowia lipolytica* lipase using polyethylene glycol (PEG) and potassium ATPS was 4 °C.

### 2.6. Lipase Recovery

**Figure 5:**
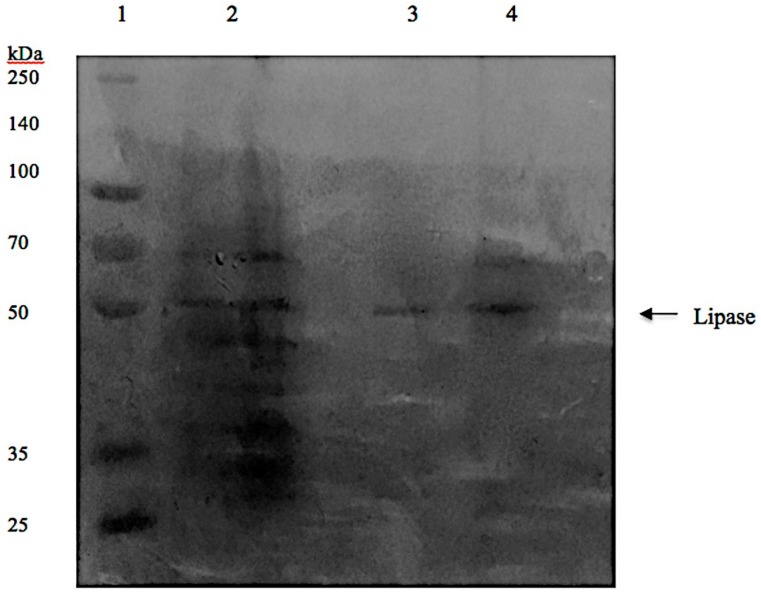
SDS-PAGE analysis of lipase recovered using Triton X-100/maltitol ATPS. The protein molecular weight of the standard protein markers ranged from 260 to 25 kDa. Lane 1: protein molecular markers, lane 2: crude feedstock, lane 3 and 4: ATPS top phase.

The optimum recovery of lipase from crude load was achieved in Triton X-100/maltitol ATPS with the TLL of 46.4% (*w*/*w*), 20% crude feedstock, pH 8 and temperature of 30 °C. The SDS-PAGE profile of the purified lipase from *L. mesenteroides* subsp. *mesenteroides* ATCC 8293 was depicted in Figure 5. In Lane 1, Spectra™ Multicolor Broad Range Protein Ladder 26634 (Thermo Fisher Scientific, Waltham, MA, USA) was used as protein marker. The supernatant containing crude lipase (Lane 2) showed multiple bands on the SDS-PAGE gel, indicating the presence of contaminant proteins in the original crude feedstock. The sample obtained from the top phase with the highest lipase activity was presented in Lane 3 and 4 and it was revealed that the molecular weight of the purified lipase was approximately 50 kDa as the presence of a band corresponding to the protein marker with the aforementioned molecular weight could be seen on the SDS-PAGE gel. However, in lane 4, the appearance of another band at about 70 kDa was also observed. This band may consist of dimers or an aggregate of the lipase and is not expected to affect the lipase activity [14]. The purification of lipase from bacterial sources using ATPS composed of Triton X-100 and Maltitol demonstrated satisfactory results, in which, it showed promising capability in lipase partitioning as previously reported ATPS, as summarized in Table 3.

### 2.7. Characterization of Purified Lipase

#### 2.7.1. Effect of PH on Activity and Stability of Lipase

**Figure 6:**
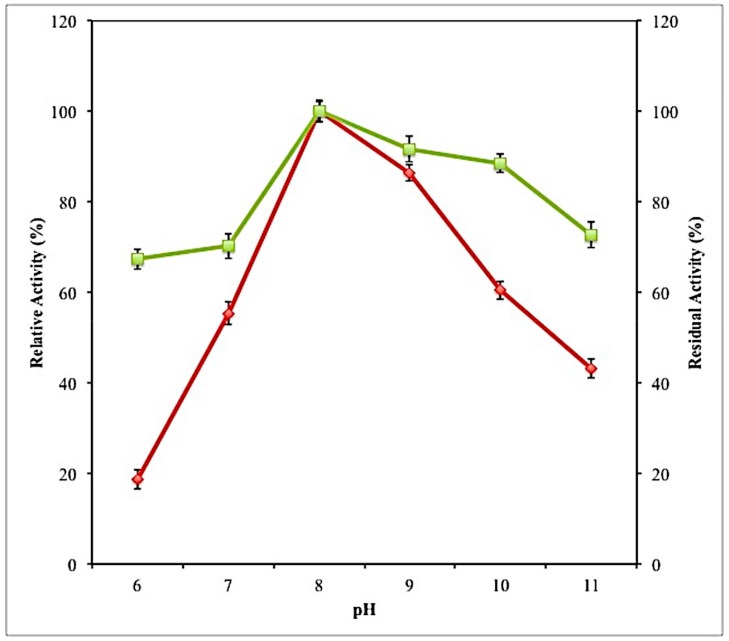
Effect of pH on the activity (♦) and stability (■) on purified lipase. Enzyme activity was determined by assaying its activity on various pH values. Enzyme stability was investigated by incubating the enzyme at different pH values (6.0–11.0) for 1 h at 37 °C. All the assays were conducted in triplicate and the average values were taken.

Lipases produced under optimized culture conditions were further characterized according to pH. pH can affect the enzyme activity as each enzyme has its own optimum pH, at which the enzyme works the best. The enzyme exhibited maximum activity at pH 8.0 (Figure 6). The optimum pH of lipase from *L. mesenteroides* subsp. *mesenteroides* ATCC 8293 was similar to the optimum pH from another lactic acid bacterium, *Lactobacillus* sp. G5 [11]. Generally, lipases produced by bacteria have neutral or alkaline pH optima. A sharp declined can be observed at pH lower than 8.0 in which lipase activity was reduced by 82% when pH was decreased to 6.0.

Enzyme activity will decline at above and below the optimum pHs. Any changes in pH will leads to the alteration of electric charges on the enzymes. Alteration in the electric charge of the enzyme affects the ionic bonds that stabilized the tertiary and quaternary structures of the enzyme. This will eventually lead to the changes in the three-dimensional conformation of the enzyme and its activity. Hence, changes in pH can reduce the effectiveness of the enzyme. The enzyme was observed to be stable at wide range of pH (6.0–11.0) as more than 65% residual activity remained after 1-h incubation at 37 °C.

#### 2.7.2. Effect of Temperature on Activity and Stability of Crude Lipase

**Figure 7:**
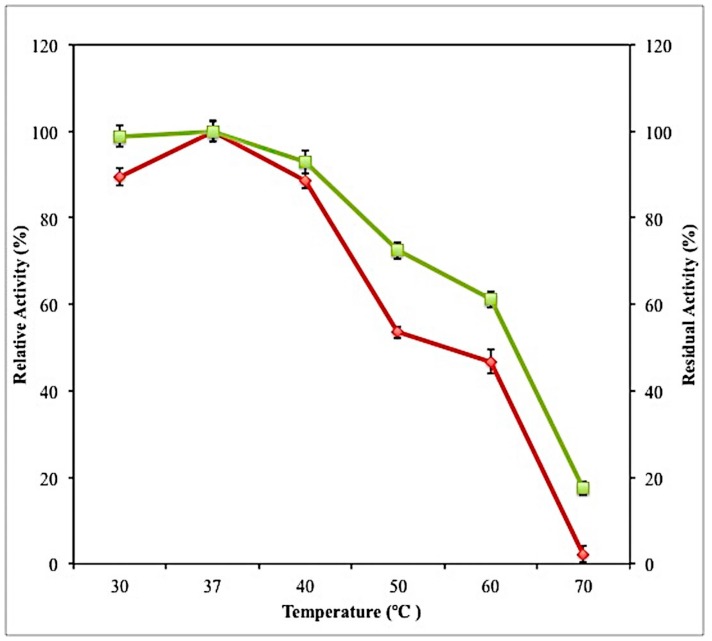
Effect of temperature on the activity (♦) and stability (■) of lipase produced by *L. mesenteroides* subsp. *mesenteroides* ATCC 8293. Enzyme activity was determined by assaying its activity at temperatures ranging from 30–70 °C Enzyme stability was investigated by incubating the enzyme at various for 1 h at pH 8, in 50 mM Tris-HCl Buffer. All the assays were conducted in triplicate and the average values were calculated.

Another factor that can affect the enzyme activity is temperature. Temperature can influence enzyme activity by altering the shape of the enzyme and altering the rate of molecular motion [19]. The enzyme was observed to be active over a wide range of temperatures (30–70 °C). Enzyme activity increased when the temperature increased from 30 °C to 37 °C (Figure 7).

The optimum temperature for lipase activity was 37 °C. The optimum temperature of this enzyme was higher than lipases from another lactic acid bacterium, which was *Lactobacillus plantarum* 2739 that has an optimum temperature of 35 °C [20]. Lipase activities decreased when the temperature increased above 37 °C. The enzyme lost 98% of its original activity at temperature 70 °C. At extreme temperature, such as 70 °C, the active sites are altered to an extent that they can no longer fit the substrate. At this point, the enzymes have been denatured, thus, enzyme activity drastically decreased [21]. The stability of the enzymes was investigated by incubating the enzymes at temperature ranging from 30 to 70 °C for 1 h. After incubation for 1 h, the enzyme retained more than 90% of its initial activity at temperature 30–40, and retained more than 60% of its initial activity after 1-h incubation at 50–60 °C.

#### 2.7.3. Effect of Surfactant Agent on Lipase Activity

Various surfactants were used in this study to investigate the effect of these additives on the activity of the purified lipase. The presence of non-ionic surfactants, which were Triton X-100 (106.92%), Tween 80 (100.21%) and Tween 20 (139.43%) were observed to induce lipase activity. However, lipase activity was inhibited in the presence of 0.1% (*w*/*v*) SDS, an ionic surfactant, as lipase activity dropped to 21.36% after 30 min of incubation (Table 4). These results were in accordance with the findings by Esakkiraj et al. [22], who revealed that Triton X-100, Tween 80 and Tween 20 enhanced lipase activity from *Bacillus* sp. PU1 by 33%, 55% and 58%, respectively. These conditions might have occurred due to the hydrophilic/lipophilic balance (HLB) of the surfactants. HLB is the way a particular detergent distributes between non-polar and polar phases. Generally, non-ionic surfactants such as Triton X-100 (HLB:13.5), Tween 80 (HLB:15) and Tween 20 (HLB:16.7) have relatively low HLB compared to anionic surfactant such as SDS, which has HLB value of 40. Surfactants with low HLB values caused less detrimental effects on lipase activity [23]. Besides, inhibition of lipase activity by SDS was due to the fact that presence of SDS leads to conformational changes of the enzyme’s active site and changes on the hydrophobicity of the enzyme surface. These will eventually result in the partial or complete unfolding of enzyme’s tertiary structure, thus, making the enzyme to be inactive [24].

#### 2.7.4. Effect of Metal Ions on Lipase Activity

The presence of metal ions can greatly influence the function and structure of the enzyme. Metal ions can alter the behavior and solubility of ionized fatty acids in the event of complex formation with metal ions. Besides, they can also influence the direct inhibition of enzyme catalytic functions when the metal ions bind with the enzyme [8]. Therefore, metal ions can either promote or inhibit enzyme activity. The effect of different metal ions on lipase activity is presented in Table 4. The metal ions from Group I (Na^+^ and K^+^) enhanced lipase activity at 5 mM concentration after 30 min of incubation at 37 °C. Metal ions from Group II (Mg^2+^ and Ca^2+^) demonstrated enhancement effects on the purified lipase at 5 mM concentration. Purified lipase treated with Mg^2+^ and Ca^2+^ exhibited an increment of 53.99% and 21.71% in their activities after 30 min of incubation. In agreement with these findings, several studies have reported that the activity of lipases from *B. subtilis* DR8806 [8], *B. subtilis* [25], *P. fluorescens* MTCC 2421 [26], and *Pseudomonas gessardii* [27] was enhanced in the presence of Mg^2+^ and K^+^ ions. Mg^2+^ can enhance lipase activity because the most lipases have negatively-charge carboxylate side-chain groups (aspartyl or glutamyl residues) in their catalytic triads. Folding of polypeptide chain brings these side-chain groups together. The Mg^2+^/Ca^2+^ bridge is responsible for the ‘cross-linked’ of the polypeptide chain, which then increases the rigidity, and stability of the enzymes. The activity of lipase produced by *L. mesenteroides* subsp. *mesenteroides* ATCC 8293 was strongly inhibited by 50% in the presence of divalent ions, which were Zn^2+^ and Cu^2+^ at 5 mM concentration. The inhibitory effect of these ions on lipase activity might be due to the fact that they negatively affect the conformation and stability of the enzymes by interacting with the charged side chain groups of accessible amino acids [28]. Enzyme inhibition by Zn^2+^ and Cu^2+^ has previously reported for lipases from *P. aeruginosa* AAU2 [23], *B. subtilis* DR8806 [8], *B. stearothermophilus* MC 7 [29] and *C. viswanathii* [24].

## 3. Materials and Methods

### 3.1. Materials

Pure culture of *Leuconostoc mesenteroides* subsp. *mesenteroides* ATCC 8293 was obtained from American Type Culture Collection (Manassas, VA, USA). De Man, Rogosa and Sharpe (MRS) agar and broth were purchased from Difco (Detroit, MI, USA). *para*-Nitrophenyl palmitate (pNPP), bovine serum albumin (BSA), sodium dodecyl sulfate (SDS), and Bradford Reagent were acquired from Sigma-Aldrich Co. (St. Louis, Mo, USA). Peptone was obtained from Oxoid (Basingstoke, UK). Gum Arabic, Triton X-100, isopropanol, Tween 80, and sodium chloride were bought from Merck (Darmstadt, Germany). Maltitol (>97 wt % pure) utilized in the ATPS was purchased from Alfa Aesar (Heysham, UK).

### 3.2. Production of Extracellular Lipase

Lipase production was performed in 250 mL Erlenmeyer flasks containing 50 mL of the production medium consisting of the following composition (g/L): peptone, 0.2% (*w*/*v*), olive oil, 1.0% (*v*/*v*), monopotassium phosphate (KH_2_PO_4_) 0.5 g, dipotassium phosphate (K_2_HPO_4_) 0.5 g, sodium chloride (NaCl) 0.1 g, calcium chloride (CaCl_2_) 0.1 g, magnesium sulfate heptahydrate (MgSO_4_.7H_2_O) 0.5 g, gum arabic, 0.1% (*w*/*v*) and tween 80, 0.1% (*v*/*v*) per liter. The medium was sterilized for 15 min at 121 °C and cooled down to room temperature. After cooling, the medium was aseptically inoculated with 4% (*v*/*v*) of the pure culture. The flasks were incubated for 72 h at 30 °C, pH 6 in an orbital shaker incubator operating at 150 rpm. After 72-h incubation, the culture broth was centrifuged using a refrigerated centrifuge (Centurion Scientific, Chichester, UK) at 10,000 rpm, 4 °C for 10 min. Only the cell-free supernatant containing the crude enzyme was collected. The collected supernatant was further used in the purification studies.

### 3.3. Binodal Curve and Tie-Lines

The binodal curve was determined by using turbidometric titration method [30]. Test tubes (10 mL) were used for the preparation of 5 g systems. The systems were developed by using previously prepared stock solutions of Triton X-100 (≈90–100 wt %) and maltitol (≈40–80 wt %). The mixture of these two components produced turbid solution indicating the formation of two phases. The weight of the test tube containing both Triton X-100 and maltitol was recorded. Then repetitive drop-wise addition of distilled water to the systems containing Triton X-100 and maltitol of known weights was carried out until the solution turned clear, indicating the formation of one phase. The system was then centrifuged for 5 min at 2000× *g* to confirm that one-phase system has formed.

The determination of the tie lines was conducted based on the methods described by Merchuk et al. [13]. Using a simple gravimetric method, a mixture of Triton X-100 and maltitol in the two-phase region of the phase diagram was prepared. The mixture was then stirred thoroughly and left overnight at room temperature to allow the occurrence of complete separation into top and bottom phases. The top and bottom phases were then taken out separately and weighted. The tie lines are correlated with the binodal curve as in Equation (1):

(1) $$\left[\mathrm{Triton}X-100]=A\exp\{(B\times [\mathrm{Maltitol}{]}^{0.5}\right)-(C\times {\left[\mathrm{Maltitol}\right]}^{3})\},$$

where A, B and C are constant parameters obtained by the regression of the experimental binodal data.

The determination of the TLs was achieved by solving the following Equations (2)–(5) for the unknown values of [Triton X-100]_T_, [Triton X-100]_B_, [Maltitol]_T_ and [Maltitol]_B_:

(2) $${\left[\mathrm{Triton}X-100\right]}_{T}=A\exp\{(B\times [\mathrm{Maltitol}{]}_{T}^{0.5})-\left(C\times {\left[\mathrm{Maltitol}\right]}_{T}^{3})\right\},$$

(3) $${\left[\mathrm{Triton}X-100\right]}_{B}=A\exp\{(B\times [\mathrm{Maltitol}{]}_{B}^{0.5})-\left(C\times {\left[\mathrm{Maltitol}\right]}_{B}^{3})\right\},$$

(4) $${\left[\mathrm{Triton}X-100\right]}_{T}={\left([\mathrm{Triton}X-100\right]}_{M}/\alpha )-\left((1-\alpha )/\alpha \right){\left[\mathrm{Triton}X-100\right]}_{B},$$

(5) $${\left[\mathrm{Maltitol}\right]}_{T}={\left([\mathrm{Maltitol}\right]}_{M}/\alpha )-\left((1-\alpha )/\alpha \right){\left[\mathrm{Maltitol}\right]}_{B},$$

where the subscripts M, T and B represent the initial mixture, the top and the bottom phases, respectively. The value of α is the ratio between the mass of the top phase and the total weight of the system.

The respective TLLs were determined using the following Equation (6):

(6) $$\mathrm{TLL}=\sqrt{{\left({\left[\mathrm{Maltitol}\right]}_{T}-{\left[\mathrm{Maltitol}\right]}_{B}\right)}^{2}+{\left({\left[\mathrm{Tri}X-100\right]}_{T}‒{\left[\mathrm{Tri}X-100\right]}_{B}\right)}^{2}},$$

### 3.4. Lipase Partitioning Experiments

For the partitioning of lipases, different phase systems were prepared in 15 mL graduated centrifuge tubes by weighing the appropriate amounts of Triton X-100, maltitol, crude feedstock and distilled water, producing systems with total weight of 10 g. The systems were mixed gently to achieve equilibration and subsequently centrifuged at 2000× *g* for 15 min to expedite phase separation. The top and bottom phases were taken out separately when the phase separation was completed. The top phase was drawn out using a pipette whereas the bottom phase was taken out using a long needle syringe. The volumes of both top and bottom phases were measured individually. Then, the lipase activity and protein concentration of the collected samples were analyzed.

### 3.5. Determination of Lipase Activity and Protein Concentration

Lipase activity in the supernatant was assayed using *p*-nitrophenyl palmitate (pNPP) as substrate. The cell-free supernatant (0.1 mL) was added into 0.9 mL of substrate mixture containing solution A (3 mg of pNPP in 1 mL isopropanol) and solution B (10 mg of gum arabic and 40 mg of Triton-X in 9 mL of 50 mM Tris-HCl buffer pH 8). The absorbance was measured spectrophotometrically at 410 nm after 30 min incubation at 37 °C in a shaking water bath [31]. One unit of activity (U) was defined as the amount of enzyme that liberated 1 μmol of *p*-nitrophenol/min under the assay conditions. All the assays were conducted in triplicate and the average values were calculated. Protein concentration was determined using bovine serum albumin as standard as described by Bradford [32].

### 3.6. Evaluation of Lipase Partitioning

The evaluation of the lipase partitioning experiments was achieved based on the calculations of several parameters. The following parameters were calculated according to Show [14] and de Brito Cardoso et al. [6]. The partition coefficient of lipase (K) was calculated based on Equation (7):

(7) $$K=\frac{A_{T}}{A_{B}},$$

where A_T_ and A_B_ were lipase activity (U/mL) in top and bottom phases, respectively.

The specific activity of the lipase (SA) was calculated using Equation (8):

(8) $$\mathrm{SA}(U/\mathrm{mg})=\frac{\mathrm{Enzyme}\;\mathrm{activity}\left(U\right)}{\mathrm{Total}\;\mathrm{protein}\left(\mathrm{mg}\right)},$$

Selectivity (*S*) was obtained using Equation (9):

(9) $$S=\frac{K_{e}}{K_{p}},$$

where *K_e_* is the enzyme partition coefficient meanwhile *K_p_* is the protein partition coefficient.

Volume ratio (V_R_) was determined according to Equation (10):

(10) $$V_{R}=\frac{V_{T}}{V_{B}},$$

where V_T_ is the volume of the top phase whereas V_B_ is the volume of the bottom phase. Purification factor (PF) was calculated as the ratio of lipase specific activity in the top phase to the initial lipase specific activity in the crude feedstock before partitioning:

(11) $$\mathrm{PF}=\frac{\mathrm{SA}\;\mathrm{of}\;\mathrm{phase}\;\mathrm{sample}}{\mathrm{SA}\;\mathrm{of}\;\mathrm{crude}\;\mathrm{feedstock}},$$

The recovery yield of lipase (%) in the top phase was determined based on Equation (12):

(12) $$\mathrm{YT}(\%)=\frac{100}{1+\left[\frac{1}{V_{R}\times K}\right]},$$

### 3.7. Sodium Dodecylsulfate-Polyacrylamide Gel Electrophoresis (SDS-PAGE) Analysis

SDS-PAGE analysis was performed using an omniPAGE Mini CVS10D vertical electrophoresis unit (Cleaver Scientific, Warwickshire, UK). The electrophoresis was conducted on a 4% polyacrylamide stacking gel as well as a 10% polyacrylamide-resolving gel and run at 120 V in Tris-glycine running buffer. The gel was then stained with Coomassie Brilliant Blue R-250. For the destaining of the gel, a destaining solution consisting 30% (*v*/*v*) methanol and 10% (*v*/*v*) was used.

### 3.8. Characterization of Purified Lipase

#### 3.8.1. Effect of Temperature on Lipase Activity and Stability

The optimum temperature of lipase activity was determined by measuring enzyme activity at various temperatures ranging from 30 °C to 70 °C for 30 min in Tris-HCl buffer, pH 8. The influence of temperature on lipase stability was investigated by pre-incubating the enzyme for 1 h in 50 mM Tris-HCl buffer, pH 8 at the above-mentioned temperature. The residual enzyme activity was measured at 37 °C, according to the standard assay conditions.

#### 3.8.2. Effect of PH on Lipase Activity and Stability

The optimum pH of the lipase activity was investigated by assaying its activity in a pH range from 6.0 to 11.0 in various buffers at 50 mM concentration: potassium phosphate buffer (pH 6.0–7.0), Tris-HCl buffer (pH 8.0–9.0), and glycine-NaOH buffer (pH 10.0–11.0). The pH stability of the enzyme was determined by pre-incubating the enzyme in the pH ranging from 6–11, for 1 h at 37 °C. The residual lipase activity was analyzed according to standard assay conditions.

#### 3.8.3. Effect of Metal Ions and Surfactants on Lipase Activity

The effect of various metal ions (Mg^2+^, K^+^, Na^+^, Ca^2+^, Cu^2+^, Fe^2+^, Zn^2+^; 5 mM) and surfactants (Triton X-100, Tween 80, Tween 20 and SDS) were investigated by pre-incubating the purified lipase with the aforementioned additives for 30 min at 37 °C in 50 mM Tris-HCl buffer, pH 8. After incubation, the relative activity was measured according to standard lipase assay condition using p-NPP as substrate.

## 4. Conclusions

To the best of author’s knowledge, this work is the first study to investigate and develop the purification method using ATPS composed of Triton X-100/Maltitol. High yield of lipase (94.7%) with a purification factor of 17.28 was successfully achieved and recovered from the Triton X-100-rich top phase when the system was incubated at pH 8, 30 °C, using 20% (*w*/*w*) crude feedstock and 46.4% (*w*/*w*) TLL. Hence, this work demonstrated that the new developed ATPS composed of Triton X-100 and maltitol is a promising method for the separation and purification of biomaterials since it reduced the loss of enzyme and eventually enhanced enzyme recovery. This is advantageous to the enzyme-related industries as it is an inexpensive and relatively straightforward purification technique since it involves only a single step. Furthermore, enzyme characterization studies showed that lipase from *L. mesenteroides* subsp. *mesenteroides* ATCC 8293 is an alkaline lipase. It is also relatively stable in the pH range of 6–11 and temperature range of 30–60 °C. The presence of metal ions from Group I and II, which were, Na^+^, K^+^, Mg^2+^ and Ca^2+^ demonstrated enhancement effect on the purified lipase. Non-ionic surfactants promoted lipase activity whereas ionic surfactant repressed the activity of purified lipase. Thus, the properties of the purified lipase showed that it has significant potential in the biotechnology applications.

## Figures and Tables

**Table 1:** Effect of different concentrations of Triton X-100 and maltitol on lipase activity of *L. mesenteroides* subsp. *mesenteroides* ATCC 8293.

Phase Component	Concentrations (%, *w*/*w*)	Relative Activity
Triton X-100	10	107.6 ± 0.31 ^a^
	20	114.9 ± 0.19 ^b^
	30	116.2 ± 0.27 ^b^
	40	122.5 ± 0.32 ^c^
	50	101.8 ± 0.15 ^d^
	60	93.1 ± 0.20 ^e^
Maltitol	5	128.9 ± 0.64 ^a^
	10	120.3 ± 0.35 ^b^
	20	113.8 ± 0.52 ^c^
	30	105.2 ± 0.22 ^d^
	40	99.1 ± 0.27 ^e^

Lipase was incubated for 1 h at 25 °C in each phase component. The residual lipase was measured using lipase assay. Lipase activity in Tris-HCl buffer (50 mM, pH 8) was used as the control. ^a–e^ mean values followed by different letters differ significantly (*p* < 0.05).

**Table 2:** Weight fraction composition for the TLs and their respective TLLs, at the top (T) and bottom phase (B), and initial biphasic composition of the mixture (M), composed of Triton X-100 ([Tri X-100]) and maltitol ([Maltitol]) and the corresponding lipase selectivity and purification factor (PF) at the respective TLLs.

Polyol	Weight Fraction, % (*w*/*w*)								
	[Tri X-100]_M_	[Maltitol]_M_	[Tri X-100]_T_	[Maltitol]_T_	[Tri X-100]_B_	[Maltitol]_B_	TLL	Selectivity	PF
Maltitol	29.91 ± 0.04	10.05 ± 0.03	34.47 ± 0.02	7.49 ± 0.04	15.67 ± 0.01	17.62 ± 0.05	21.4	4.76	2.68
	34.79 ± 0.07	10.26 ± 0.06	43.97 ± 0.04	5.44 ± 0.09	13.05 ± 0.04	21.83 ± 0.70	35.0	6.11	4.19
	30.06 ± 0.03	15.12 ± 0.05	44.96 ± 0.07	5.27 ± 0.02	12.15 ± 0.11	24.31 ± 0.07	37.9	7.43	4.32
	35.21 ± 0.14	9.83 ± 0.08	51.78 ± 0.08	4.26 ± 0.05	11.57 ± 0.61	27.33 ± 0.22	46.4	10.25	7.41

**Table 3:** A comparison of lipase purification results of previously reported ATPS with the purification results of the present work.

Phase-Forming Components	Bacterial Sources	Yield (%)	PF	Reference
PEG 8000/Na_2_HPO_4_	*Enterococcus faecium* MTCC5695	82.09	5.99	[17]
PPG 400/[Ch][BES]	*Burkholderia cepacia* ST8	99.3	17.96	[4]
PEG6000 + K_2_HPO_4_/KH_2_PO_4_ + NaCl	*Burkholderia pseudomallei*	93.0	12.4	[18]
EOPO 3900/KH_2_PO_4_	*Burkholderia cenocepacia* ST8	99.0	14.0	[14]
Triton X-100/Maltitol	*L.mesenteroides* subsp. *mesenteroides* ATCC 8293	94.7	17.28	This work

**Table 4:** Effects of different additives on lipase activity.

		Relative Activity (%)
Control		100 ± 0.00 ^a^
Surfactants	Triton X-100	106.92 ± 0.05 ^b^
	Tween 20	139.43 ± 0.04 ^c^
	Tween 80	100.21 ± 0.09 ^a^
	SDS	21.36 ± 0.03 ^d^
Metal ions	Mg^2+^	153.99 ± 0.05 ^e^
	K^+^	141.72 ± 0.04 ^f^
	Ca^2+^	121.71 ± 0.05 ^g^
	Na^+^	105.22 ± 0.07 ^h^
	Zn^2+^	54.43 ± 0.04 ^i^
	Cu^2+^	53.16 ± 0.06 ^i^

The sample sizes for all experiments were three. ^a–i^ mean values followed by different letters differ significantly (*p* < 0.05).
